# Supplementary figures and images for: New deep learning method for efficient extraction of small water from remote sensing images
Source: PLoS One. 2022 Aug 5;17(8):e0272317. doi: 10.1371/journal.pone.0272317 (PMC9355223; doi:10.1371/journal.pone.0272317)

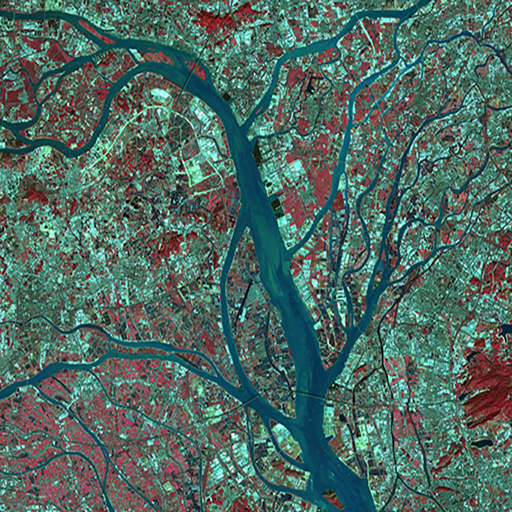

Supplement: S1 File — All codes used in this project are included in the S1_File.zip. (ZIP) [file pone.0272317.s001.zip › code/deeplab/results/0_image.png]

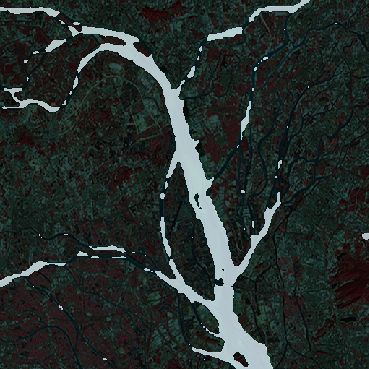

Supplement: S1 File — All codes used in this project are included in the S1_File.zip. (ZIP) [file pone.0272317.s001.zip › code/deeplab/results/0_overlay.png]

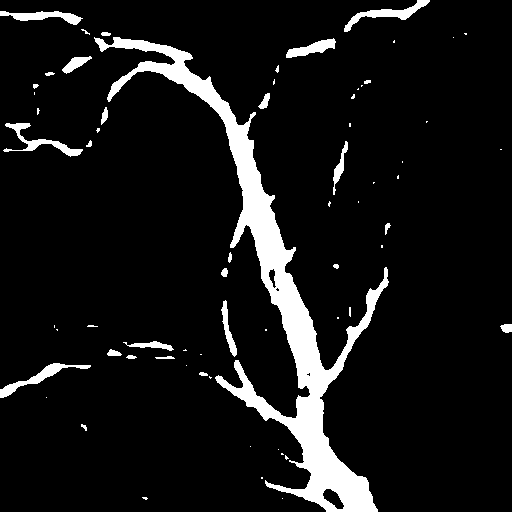

Supplement: S1 File — All codes used in this project are included in the S1_File.zip. (ZIP) [file pone.0272317.s001.zip › code/deeplab/results/0_pred.png]

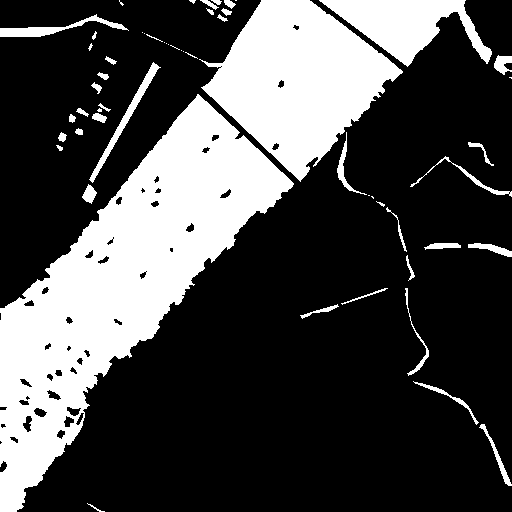

Supplement: S1 File — All codes used in this project are included in the S1_File.zip. (ZIP) [file pone.0272317.s001.zip › code/deeplab/results/0_target.png]

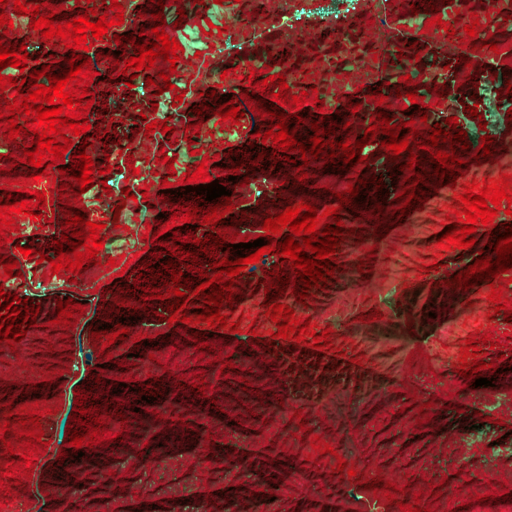

Supplement: S1 File — All codes used in this project are included in the S1_File.zip. (ZIP) [file pone.0272317.s001.zip › code/deeplab/results/1969_image.png]

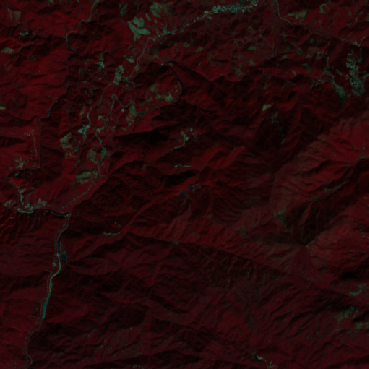

Supplement: S1 File — All codes used in this project are included in the S1_File.zip. (ZIP) [file pone.0272317.s001.zip › code/deeplab/results/1969_overlay.png]

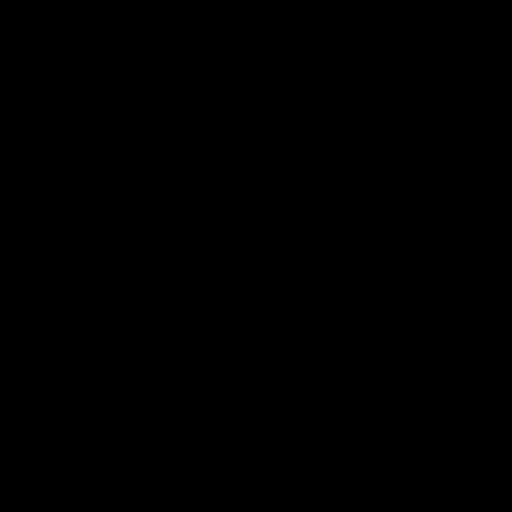

Supplement: S1 File — All codes used in this project are included in the S1_File.zip. (ZIP) [file pone.0272317.s001.zip › code/deeplab/results/1969_pred.png]

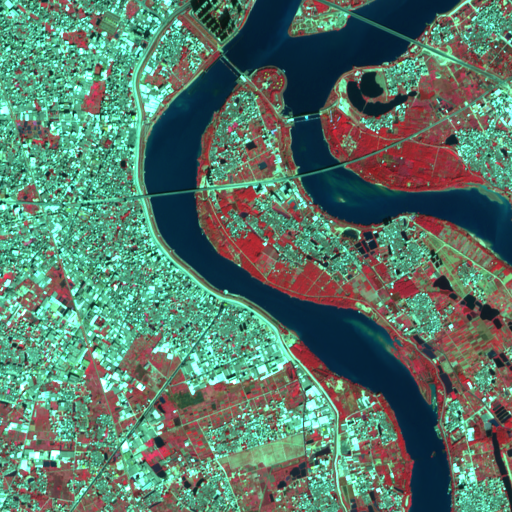

Supplement: S1 File — All codes used in this project are included in the S1_File.zip. (ZIP) [file pone.0272317.s001.zip › code/deeplab/results/1970_image.png]

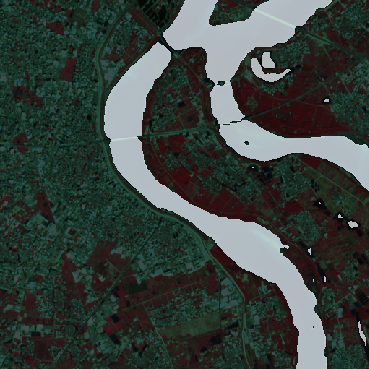

Supplement: S1 File — All codes used in this project are included in the S1_File.zip. (ZIP) [file pone.0272317.s001.zip › code/deeplab/results/1970_overlay.png]

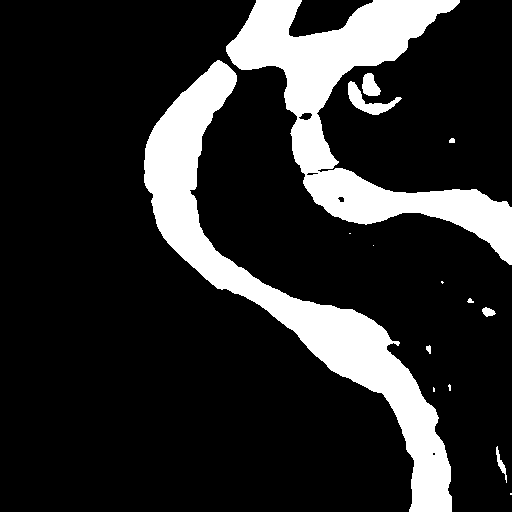

Supplement: S1 File — All codes used in this project are included in the S1_File.zip. (ZIP) [file pone.0272317.s001.zip › code/deeplab/results/1970_pred.png]

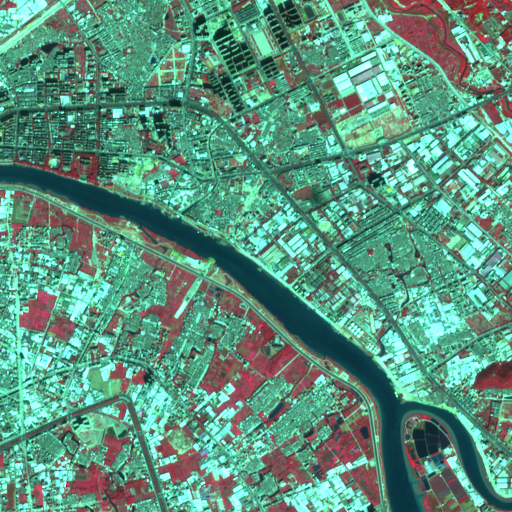

Supplement: S1 File — All codes used in this project are included in the S1_File.zip. (ZIP) [file pone.0272317.s001.zip › code/deeplab/results/1971_image.png]

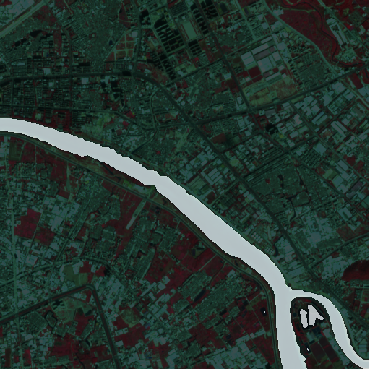

Supplement: S1 File — All codes used in this project are included in the S1_File.zip. (ZIP) [file pone.0272317.s001.zip › code/deeplab/results/1971_overlay.png]

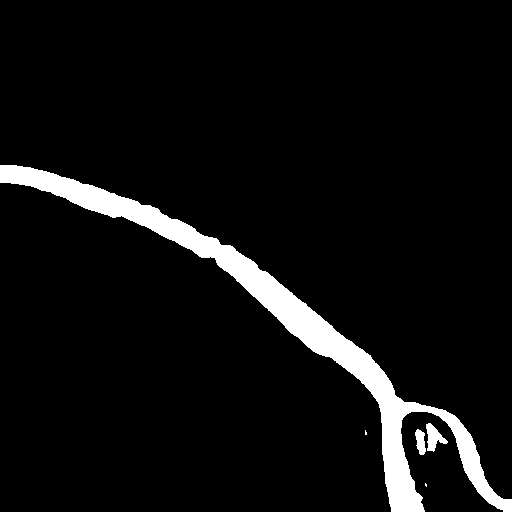

Supplement: S1 File — All codes used in this project are included in the S1_File.zip. (ZIP) [file pone.0272317.s001.zip › code/deeplab/results/1971_pred.png]

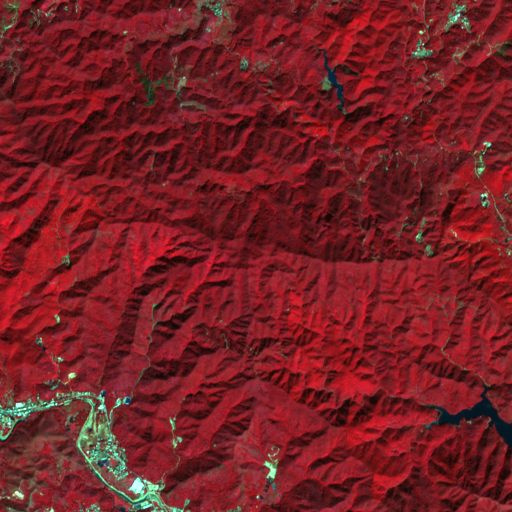

Supplement: S1 File — All codes used in this project are included in the S1_File.zip. (ZIP) [file pone.0272317.s001.zip › code/deeplab/results/1972_image.png]

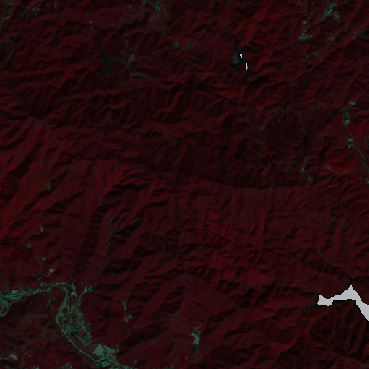

Supplement: S1 File — All codes used in this project are included in the S1_File.zip. (ZIP) [file pone.0272317.s001.zip › code/deeplab/results/1972_overlay.png]

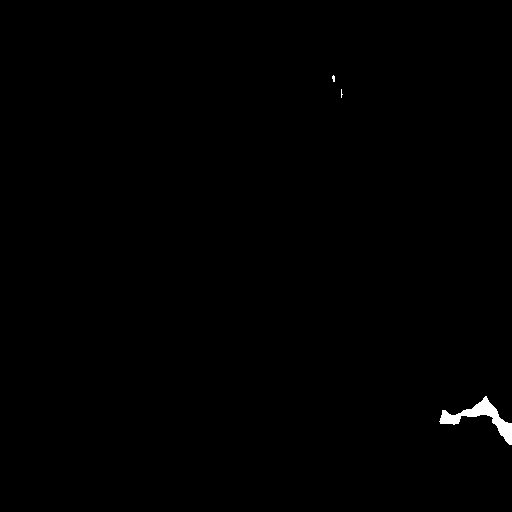

Supplement: S1 File — All codes used in this project are included in the S1_File.zip. (ZIP) [file pone.0272317.s001.zip › code/deeplab/results/1972_pred.png]

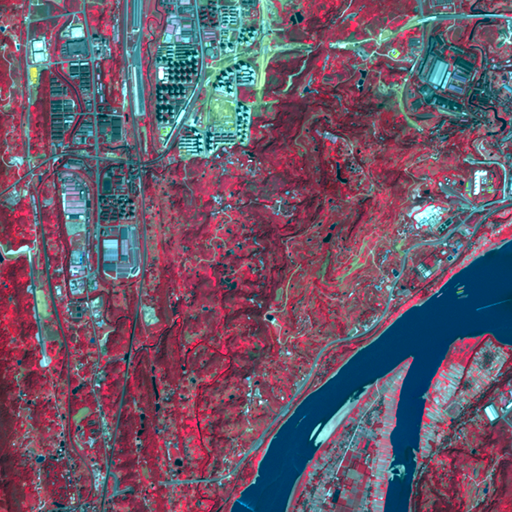

Supplement: S1 File — All codes used in this project are included in the S1_File.zip. (ZIP) [file pone.0272317.s001.zip › code/deeplab/results/1973_image.png]

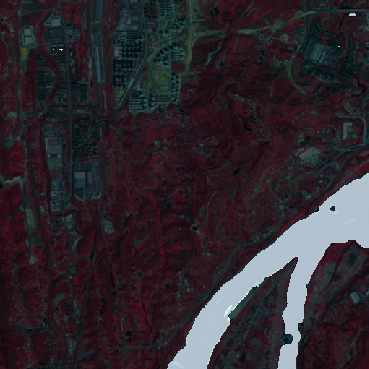

Supplement: S1 File — All codes used in this project are included in the S1_File.zip. (ZIP) [file pone.0272317.s001.zip › code/deeplab/results/1973_overlay.png]

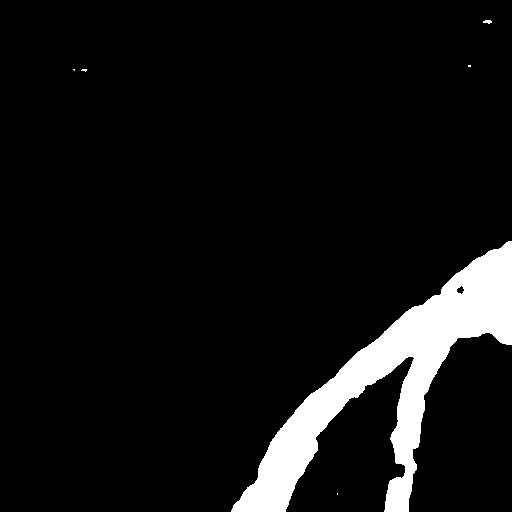

Supplement: S1 File — All codes used in this project are included in the S1_File.zip. (ZIP) [file pone.0272317.s001.zip › code/deeplab/results/1973_pred.png]

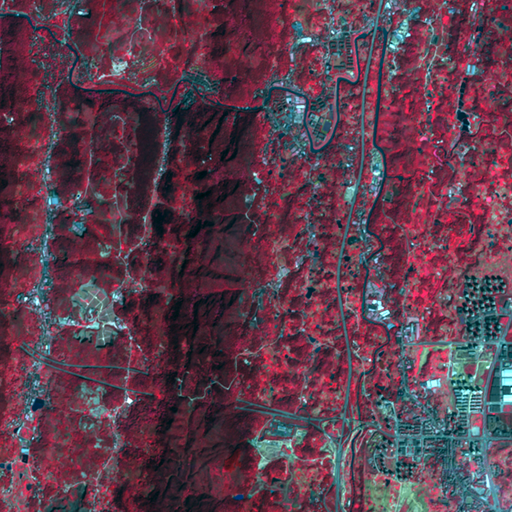

Supplement: S1 File — All codes used in this project are included in the S1_File.zip. (ZIP) [file pone.0272317.s001.zip › code/deeplab/results/1974_image.png]

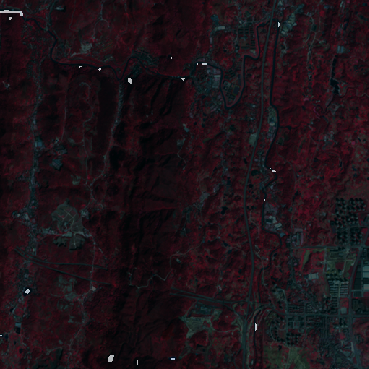

Supplement: S1 File — All codes used in this project are included in the S1_File.zip. (ZIP) [file pone.0272317.s001.zip › code/deeplab/results/1974_overlay.png]

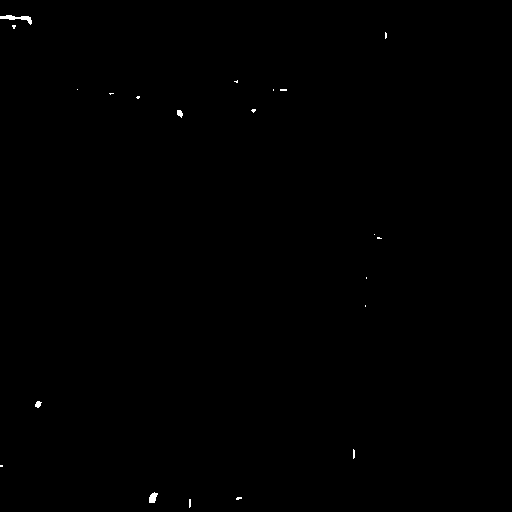

Supplement: S1 File — All codes used in this project are included in the S1_File.zip. (ZIP) [file pone.0272317.s001.zip › code/deeplab/results/1974_pred.png]

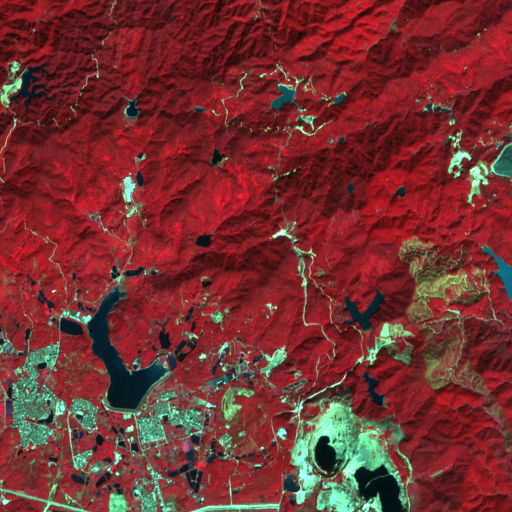

Supplement: S1 File — All codes used in this project are included in the S1_File.zip. (ZIP) [file pone.0272317.s001.zip › code/deeplab/results/1975_image.png]

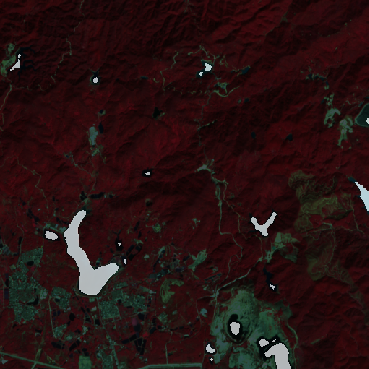

Supplement: S1 File — All codes used in this project are included in the S1_File.zip. (ZIP) [file pone.0272317.s001.zip › code/deeplab/results/1975_overlay.png]

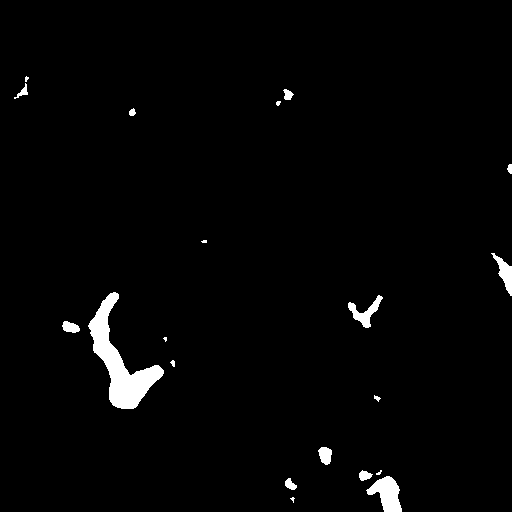

Supplement: S1 File — All codes used in this project are included in the S1_File.zip. (ZIP) [file pone.0272317.s001.zip › code/deeplab/results/1975_pred.png]

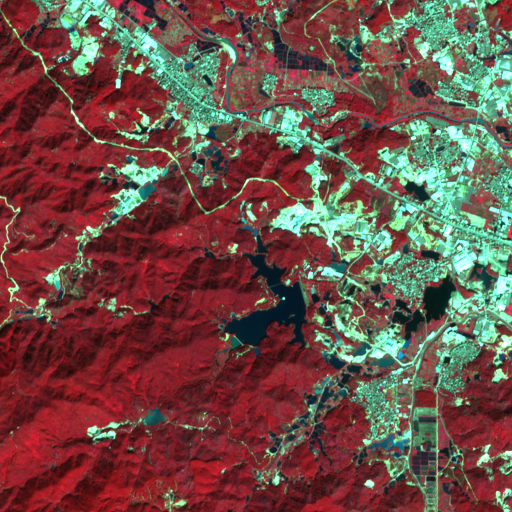

Supplement: S1 File — All codes used in this project are included in the S1_File.zip. (ZIP) [file pone.0272317.s001.zip › code/deeplab/results/1976_image.png]

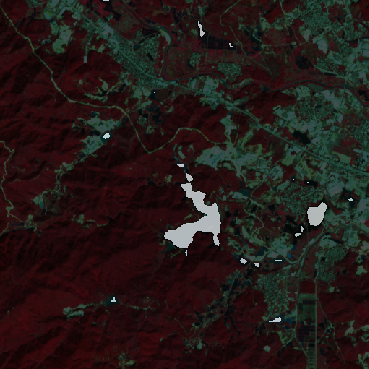

Supplement: S1 File — All codes used in this project are included in the S1_File.zip. (ZIP) [file pone.0272317.s001.zip › code/deeplab/results/1976_overlay.png]

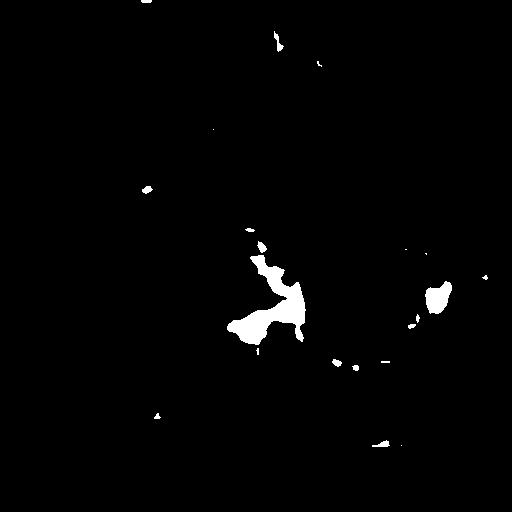

Supplement: S1 File — All codes used in this project are included in the S1_File.zip. (ZIP) [file pone.0272317.s001.zip › code/deeplab/results/1976_pred.png]

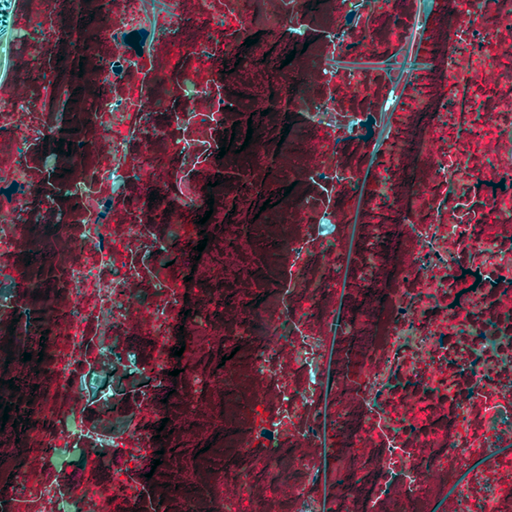

Supplement: S1 File — All codes used in this project are included in the S1_File.zip. (ZIP) [file pone.0272317.s001.zip › code/deeplab/results/1977_image.png]

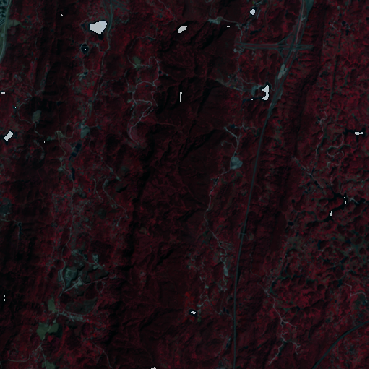

Supplement: S1 File — All codes used in this project are included in the S1_File.zip. (ZIP) [file pone.0272317.s001.zip › code/deeplab/results/1977_overlay.png]

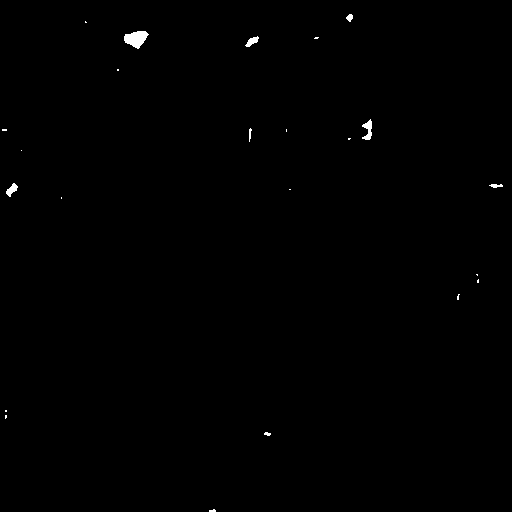

Supplement: S1 File — All codes used in this project are included in the S1_File.zip. (ZIP) [file pone.0272317.s001.zip › code/deeplab/results/1977_pred.png]

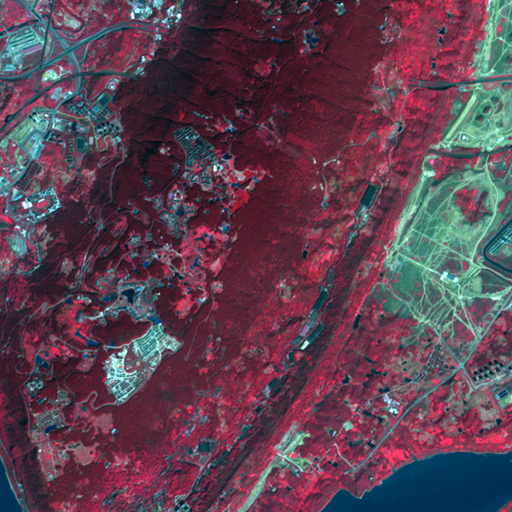

Supplement: S1 File — All codes used in this project are included in the S1_File.zip. (ZIP) [file pone.0272317.s001.zip › code/deeplab/results/1978_image.png]

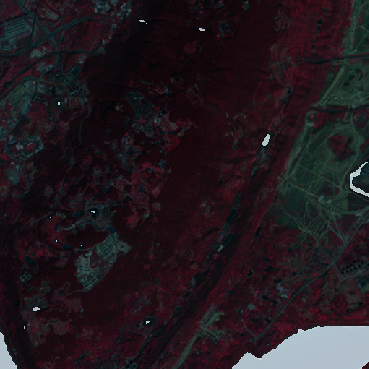

Supplement: S1 File — All codes used in this project are included in the S1_File.zip. (ZIP) [file pone.0272317.s001.zip › code/deeplab/results/1978_overlay.png]

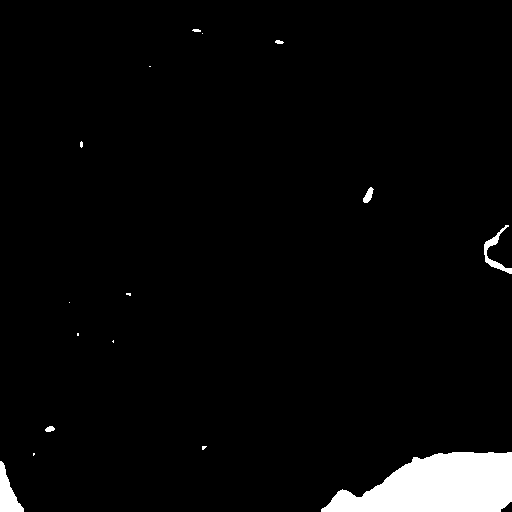

Supplement: S1 File — All codes used in this project are included in the S1_File.zip. (ZIP) [file pone.0272317.s001.zip › code/deeplab/results/1978_pred.png]

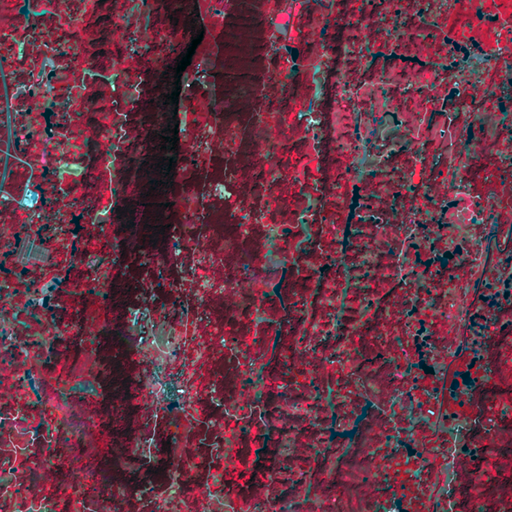

Supplement: S1 File — All codes used in this project are included in the S1_File.zip. (ZIP) [file pone.0272317.s001.zip › code/deeplab/results/1979_image.png]

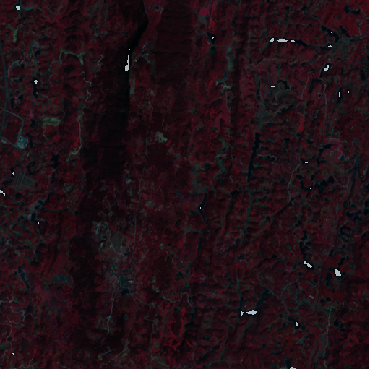

Supplement: S1 File — All codes used in this project are included in the S1_File.zip. (ZIP) [file pone.0272317.s001.zip › code/deeplab/results/1979_overlay.png]

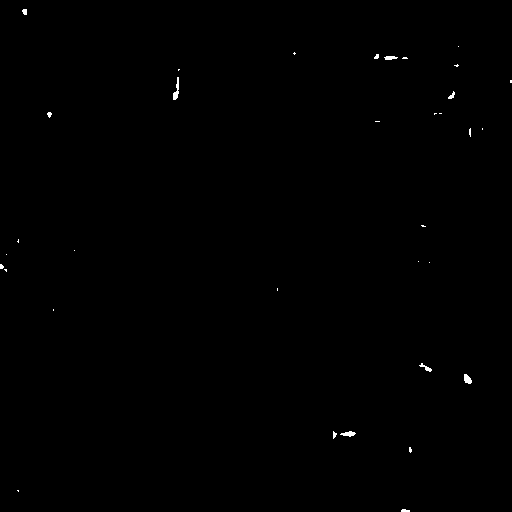

Supplement: S1 File — All codes used in this project are included in the S1_File.zip. (ZIP) [file pone.0272317.s001.zip › code/deeplab/results/1979_pred.png]

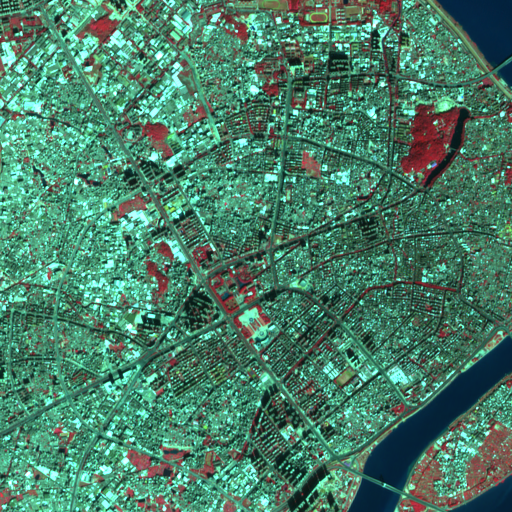

Supplement: S1 File — All codes used in this project are included in the S1_File.zip. (ZIP) [file pone.0272317.s001.zip › code/deeplab/results/1980_image.png]

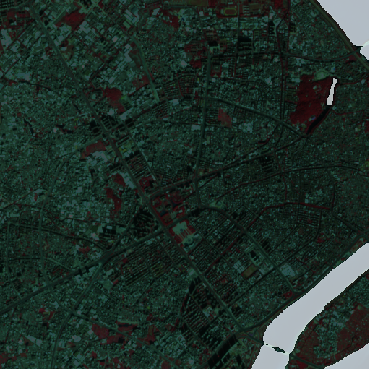

Supplement: S1 File — All codes used in this project are included in the S1_File.zip. (ZIP) [file pone.0272317.s001.zip › code/deeplab/results/1980_overlay.png]

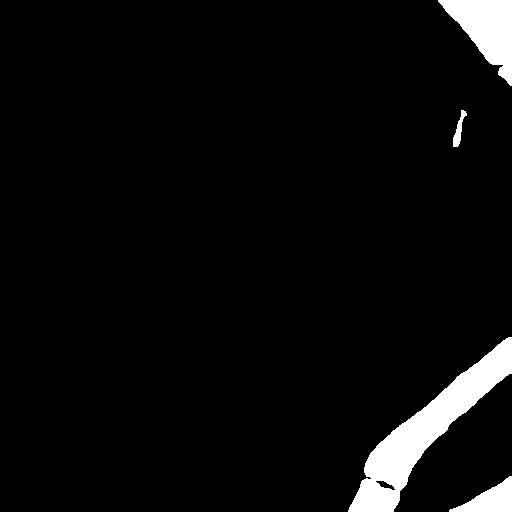

Supplement: S1 File — All codes used in this project are included in the S1_File.zip. (ZIP) [file pone.0272317.s001.zip › code/deeplab/results/1980_pred.png]

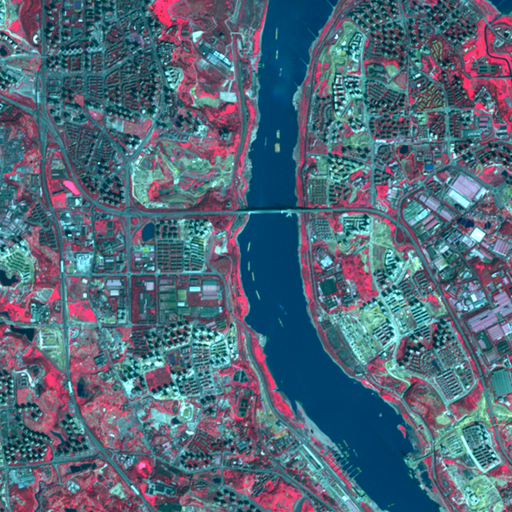

Supplement: S1 File — All codes used in this project are included in the S1_File.zip. (ZIP) [file pone.0272317.s001.zip › code/deeplab/results/1981_image.png]

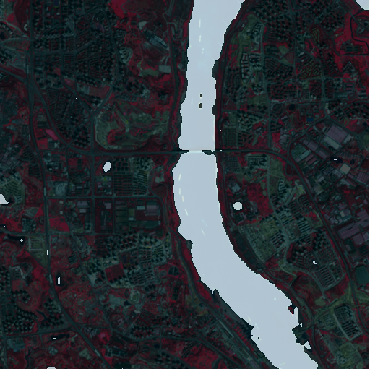

Supplement: S1 File — All codes used in this project are included in the S1_File.zip. (ZIP) [file pone.0272317.s001.zip › code/deeplab/results/1981_overlay.png]

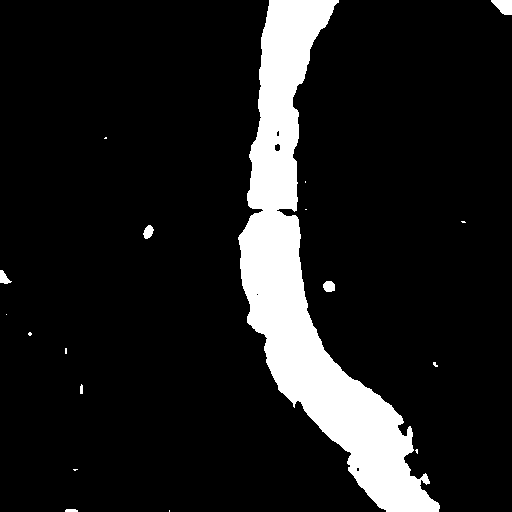

Supplement: S1 File — All codes used in this project are included in the S1_File.zip. (ZIP) [file pone.0272317.s001.zip › code/deeplab/results/1981_pred.png]

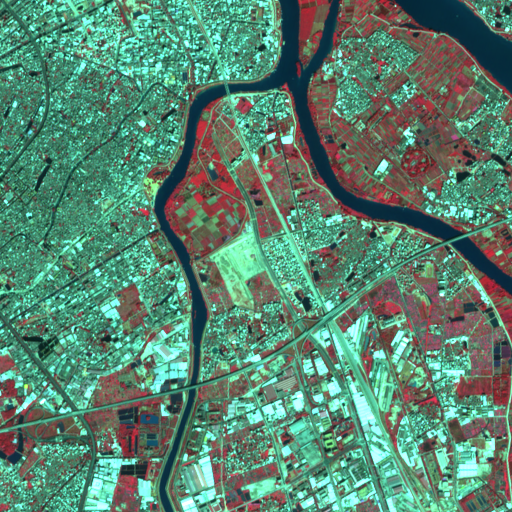

Supplement: S1 File — All codes used in this project are included in the S1_File.zip. (ZIP) [file pone.0272317.s001.zip › code/deeplab/results/1982_image.png]

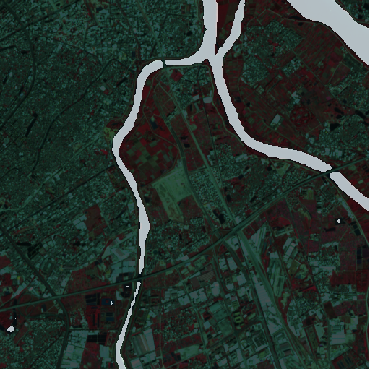

Supplement: S1 File — All codes used in this project are included in the S1_File.zip. (ZIP) [file pone.0272317.s001.zip › code/deeplab/results/1982_overlay.png]

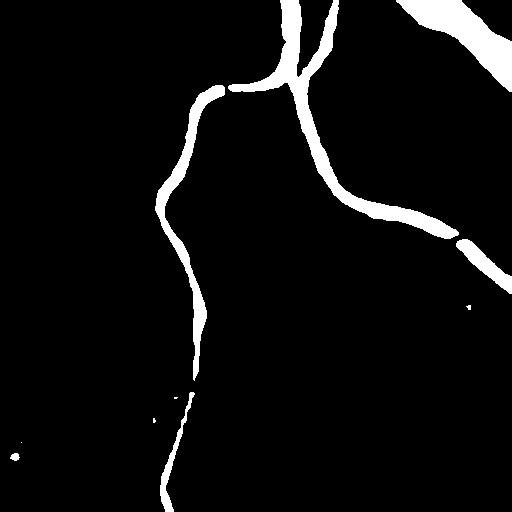

Supplement: S1 File — All codes used in this project are included in the S1_File.zip. (ZIP) [file pone.0272317.s001.zip › code/deeplab/results/1982_pred.png]

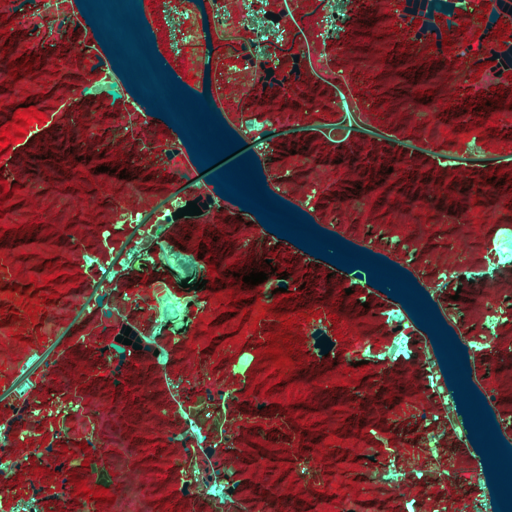

Supplement: S1 File — All codes used in this project are included in the S1_File.zip. (ZIP) [file pone.0272317.s001.zip › code/deeplab/results/1983_image.png]

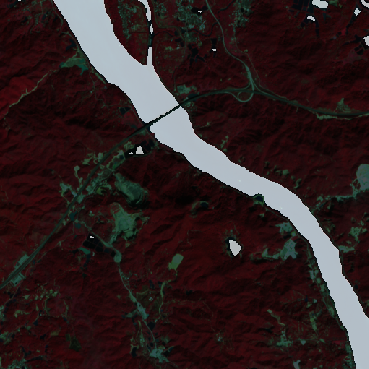

Supplement: S1 File — All codes used in this project are included in the S1_File.zip. (ZIP) [file pone.0272317.s001.zip › code/deeplab/results/1983_overlay.png]

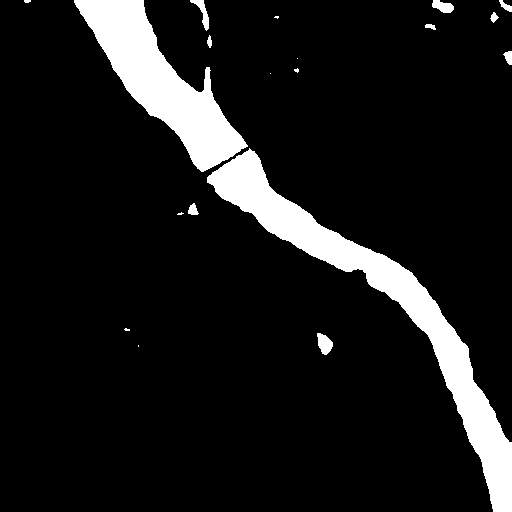

Supplement: S1 File — All codes used in this project are included in the S1_File.zip. (ZIP) [file pone.0272317.s001.zip › code/deeplab/results/1983_pred.png]

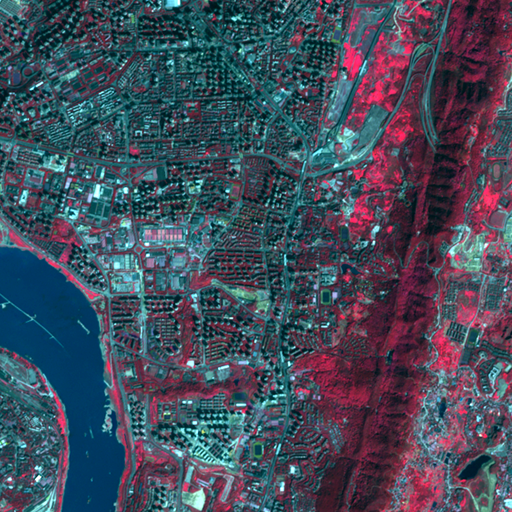

Supplement: S1 File — All codes used in this project are included in the S1_File.zip. (ZIP) [file pone.0272317.s001.zip › code/deeplab/results/1984_image.png]

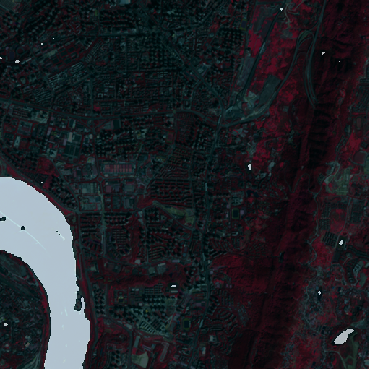

Supplement: S1 File — All codes used in this project are included in the S1_File.zip. (ZIP) [file pone.0272317.s001.zip › code/deeplab/results/1984_overlay.png]
